# Supplementary material for: A Proxy Approach to Family Involvement and Neurocognitive Function in First Episode of Non-Affective Psychosis: Sex-Related Differences
Source: Healthcare (Basel). 2023 Jun 30;11(13):1902. doi: 10.3390/healthcare11131902 (PMC10340230; doi:10.3390/healthcare11131902)
Supplement: Supplementary file 1 [file healthcare-11-01902-s001.zip › healthcare-2374434-supplementary.pdf]

Conducting ANCOVA Analysis with Rank Transformation in SPSS:

Step1: Rank transformation: The dependent variable and covariates are ranked using the default settings in the SPSS RANK procedure. This process assigns ranks to the observed values, considering all cases and disregarding the grouping variable.

Step 2: Linear regression of ranks: A linear regression analysis is performed, with the ranks of the dependent variable as the outcome and the ranks of the covariates as predictors. The residuals (raw or unstandardized) are saved, ignoring the grouping factor.

Step 3: One-way ANOVA using residuals: An ANOVA is conducted using the saved residuals from the previous regression as the dependent variable, while the grouping variable serves as the factor. The resulting F statistic from this ANOVA represents the test statistic used in Quade's analysis.
